# The role of estrogen and progesterone receptors in the rotator cuff disease: a retrospective cohort stud﻿y

**DOI:** 10.1186/s12891-021-04778-5

**Published:** 2021-10-20

**Authors:** Umile Giuseppe Longo, Alessandro Mazzola, Simone Carotti, Maria Francesconi, Simone Catapano, Francesco Magrì, Giuseppe Perrone, Sergio Morini, Sergio De Salvatore, Vincenzo Denaro

**Affiliations:** 1 Department of Orthopaedic and Trauma Surgery, Campus Bio-Medico University, Via Alvaro del Portillo 200, Trigoria, 00128 Rome, Italy; 2 Unit of Microscopic and Ultrastructural Anatomy, University Campus Bio-Medico, Rome, Italy; 3 Department of Human Pathology, University Campus Bio-Medico, Rome, Italy

**Keywords:** Estrogen, Progesterone, Receptor, Rotator, Cuff, Tendinopathy, Immunohistochemistry

## Abstract

**Background:**

Rotator cuff (RC) tears represent a common cause of shoulder pain and dysfunction in adults. The disease affects primarily women and occurs mainly in the postmenopausal period.

This study aimed to investigate immunohistochemically the presence of estrogen receptor-alpha (ER-⍺), estrogen receptor-beta (ER-β) and progesterone receptor (PR) in the supraspinatus tendon of patients with RC tendinopathy, searching for gender differences of expression. A secondary aim was to evaluate potential links between their expression and the typical histopathological findings of the ailment.

**Methods:**

Biopsies of the supraspinatus tendon were collected intraoperatively from 15 postmenopausal women and 9 men undergoing RC surgery. Specimens were stained with Haematoxylin/Eosin, Masson-Goldner Trichrome, Alcian Blu and immunohistochemical stainings for ER-⍺, ER-β and PR were performed. Tendon alterations were evaluated with the Bonar histopathological scale. Statistical tests used in this study were the Spearman correlation coefficient and the Mann-Whitney *U* test.

**Results:**

In the supraspinatus tendon, cells expressed ER-⍺ (*p* = 0.043), ER-β (*p* = 0.048) and PR (*p* = 0.004) with statistically significant differences related to age and sex of patients. Immunoreactivity was seen in the nuclei of tenocytes and vascular cells. Postmenopausal women’s samples showed a markedly higher expression of these receptors compared to their male counterpart. There was a positive correlation between the expression of ER-⍺ and ER-β (*r* = 0.59; *p* = 0.02) and between ER-β and PR (*r* = 0.72; *p* = 0.002) in women’s samples. Furthermore, in postmenopausal women the PR expression decreased with age (*r* = − 0.56; *p* = 0.027). Only in women, the ER-β expression positively correlated with the total Bonar histopathological score (*p* = 0.019) and the ER-β vascular expression positively correlated with ground substance alterations (*p* = 0.029).

**Conclusions:**

These results reveal that ERs and PR are present in the supraspinatus tendon of patients with RC tears, suggesting a role of sex hormones in the pathogenesis of the disease.

## Background

Rotator cuff (RC) disease is frequent and represents a common cause of shoulder pain, although not all patients are symptomatic [1, 2]. In the USA, each year approximately 4.5 millions of people suffer from shoulder pain and the majority of them are due to RC tears [3, 4]. The prevalence of the disease increases with age, affecting up to 67% of patients over 80 years [5, 6].

Although several risk factors have been identified, actually, the pathogenesis and the natural history of RC tendinopathy remains unclear [5, 7]. It is considered a multifactorial disease [3] with intrinsic (age, gender, genetic predisposition) and extrinsic (micro and macro-traumas) cofactors that contribute to its onset [8–10]. Recently, endocrine alterations as dysthyroidism [11], hypercholesterolemia and diabetes [12, 13] have been considered potential risk factors.

Numerous studies have indicated that the incidence of repetitive motion disorders occur primarily in females [14] and that asymptomatic full-thickness RC tears are typically diagnosed in the postmenopausal period [15]. Moreover, in women there is a higher rate of failure, in terms of functional healing, after RC surgery than men [16]. Although the reason of these gender differences remains uncertain, possible explanations include structural alterations in tendon tissue due to the exposure to oscillating levels of female sex hormones [17].

Many authors have investigated the role of female sex hormones and their receptors in the pathogenesis of some musculoskeletal disorders [18–20], including the carpal tunnel syndrome [21], the De Quervain’s Syndrome [22] and lesions of the anterior cruciate ligament [23–25].

Estrogens and progesterone are steroid hormones involved in the differentiation and development of the reproductive system, but their influence has been demonstrated also in non-reproductive systems [26]. In order to express their biological activities, female sex hormones have to interact with their specific nuclear receptors, estrogen receptor (ER) and progesterone receptor (PR). There are two different isoforms of ER: ER-⍺ and ER-β [27]; also PR presents two isoforms: PR-A and PR-B [28].

As regards the musculoskeletal system, estrogens and progesterone are known to influence fibroblast proliferation and the production of type I collagen [29]. In contrast, menopause is associated with an important reduction of type I collagen content in tendon tissue (the most important structural protein in tendons and ligaments) [30, 31]. These structural alterations could be due to low levels of sex hormones and high levels of pro-inflammatory cytokines (IL-6, TNF-⍺), that are considered physiological modifications of the postmenopausal period.

This study aimed to investigate immunohistochemically the expression of ER-⍺, ER-β, PR in the supraspinatus tendon of patients with RC tendinopathy. A secondary aim was to evaluate their possible significance in the pathogenesis of the disease.

## Methods

The study enrolled 24 patients representing a consecutive series of patients treated at the orthopedic department of our institution from November 2017 to February 2020. All the patients included in the present study presented chronic shoulder pain and functional impairment. All patients received a preoperative shoulder magnetic resonance imaging (MRI) scan in the affected side. All MRIs were performed with a 1.5-T unit. Full-thickness rotator cuff tears were measured on the sagittal (at the tuberosities) and coronal images and classified into the numbers of tendons torn: small lesions (< 1 cm of tear and <  1 tendon involved); medium lesions (1–3 cm of tear and 1 tendon involved); large (3–5 cm of tear and 2 tendons involved); massive (> 5 cm of tear and > 2 tendons involved). Partial-thickness rotator cuff tears were graded in a binary fashion as either grade 1 (less than 50% torn) or grade 2 (more than 50% torn) and according to the side torn (either articular or bursal) [32]. Primary aims of the present study were: to investigate immunohistochemically the expression of ER-⍺, ER-β, PR in the supraspinatus tendon of patients with RC tendinopathy, searching for gender differences of expression. Secondary aims of the present study were: to investigate potential links between the expression of ER-⍺, ER-β, PR and the typical histopathological tendon alterations of the RC tendinopathy (according to the Bonar scale [33, 34]). All methods were carried out in accordance with relevant guidelines and regulations. The Ethics committee of Campus Bio Medico of Rome approved the present study and all patients gave written consent to participate. Informed consent was obtained from all participants.

### Patients

None of patients included in the present study had a diagnosis of bilateral RC tendinopathy. All the women enrolled in this study were in menopause. Inclusion criteria were: clinical diagnosis of RC disease; absence of shoulder instability; absence of radiographic evidence of fractures in the humeral head and in the glenoid. Exclusion criteria were: other potential endocrine alterations implicated in the pathogenesis of rotator cuff tendinopathy (disthyroidism, hypercholesterolemia, diabetes), previous surgical treatment in the shoulder; shoulder arthritis; arthrosis of the acromioclavicular or the glenohumeral joint (Tables 1 and 2).

**Table 1:** Demographic data of the female population with RC disease

Patient number	Age at surgery	Age at Menopause	Hormone replacement therapy	Hormone suppression therapy	Body Mass Index (BMI)	Type of tendon injuries (small; medium; large; massive)	Time from injury/shoulder pain	Treatments performed before surgery
1	73	53	no	yes	29.3	Atraumatic - massive	6 years	Physical therapy
2	64	54	yes	yes	34.9	Atraumatic - massive	6 months	none
3	58	49	no	no	37.7	Atraumatic - massive	2 years	none
4	58	53	no	no	20.0	Atraumatic - medium	2 years	none
5	69	51	no	no	29.9	Atraumatic - massive	6 years	none
6	61	51	no	no	24.2	Atraumatic - large	8 months	Physical therapy
7	59	52	no	no	26.9	Traumatic - medium	1 year	none
8	74	55	no	no	26.0	Atraumatic - massive	2 years	none
9	79	50	no	no	25.4	Atraumatic - medium	5 months	none
10	74	48	no	no	26.0	Atraumatic - massive	2 years	none
11	62	50	yes	yes	27.4	Atraumatic - medium	1 year	Physical therapy
12	61	53	no	no	24.2	Traumatic - large	3 months	none
13	66	51	no	no	30.1	Atraumatic - medium	2 years	Corticosteroid injections
14	68	49	no	no	25.0	Traumatic -large	2 years	none
15	55	50	no	no	29.0	Atraumatic – medium	6 months	none

Female population: Mean age 63.93 ± 7.05; Mean age at surgery 65.41 ± 6.94; Mean age at menopause 51.27 ± 1.95; Mean BMI 27.73 ± 4.24; Mean duration of symptoms 22.67 ± 20.93 months. Full-thickness rotator cuff tears were classified into the numbers of tendons torn: small lesions (< 1 cm of tear and < 1 tendon involved); medium lesions (1–3 cm of tear and 1 tendon involved); large (3–5 cm of tear and 2 tendons involved); massive (> 5 cm of tear and > 2 tendons involved). Partial-thickness rotator cuff tears were graded in a binary fashion as either grade 1 (less than 50% torn) or grade 2 (more than 50% torn) and according to the side torn (either articular or bursal)

**Table 2:** Demographic data of the male population with RC disease

Patient number	Age at surgery	Hormonal therapy	Body Mass Index (BMI)	Type of tendon injuries	Time from injury / shoulder pain	Treatments before surgery
1	54	no	32.2	Traumatic - massive	1 year	none
2	51	no	28.0	Atraumatic - large	2 years	Corticosteroid injections
3	55	no	27.7	Traumatic - medium	2 months	none
4	77	no	26.9	Atraumatic - massive	1 year	none
5	62	no	29.7	Atraumatic - large	3 years	none
6	59	no	26.8	Traumatic - massive	1 month	none
7	66	no	29.0	Traumatic - massive	4 months	none
8	77	no	25.1	Atraumatic - medium	2 years	none
9	60	no	28.7	Atraumatic - large	5 months	Physical therapy

Male population: Mean age 61.67 ± 9.02; Mean age at surgery 62.33 ± 8.89; Mean BMI 28.23 ± 1.91; Mean duration of symptoms 13.33 ± 11.46 months

### Bioptic samples collection

**Fig. 1:**
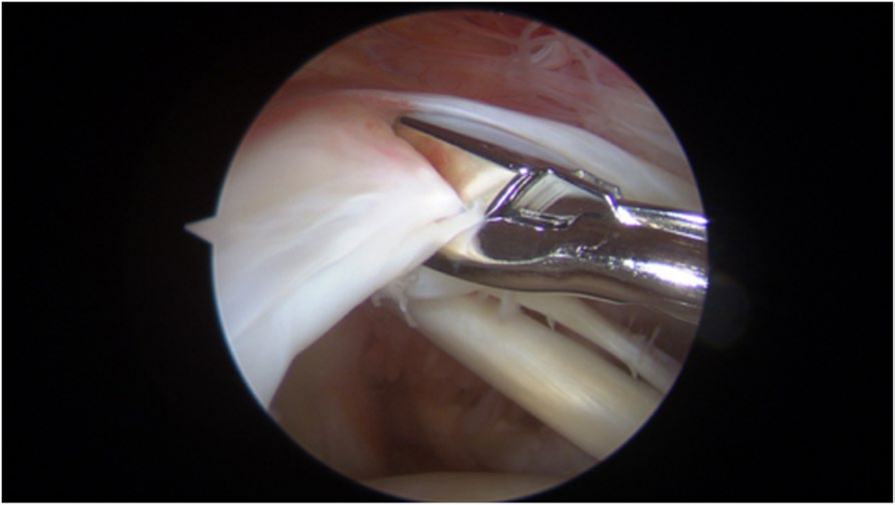
Withdrawal of the supraspinatus tendon bioptic sample during arthroscopic rotator cuff repair

Biopsies of the supraspinatus tendon were collected intraoperatively by the First Author (Fig. 1). Samples were harvested during surgery at the myotendineous junction of the pathological supraspinatus tendon by means of arthroscopic instruments.

### Histopathology and immunohistochemistry

Laboratory analysis were performed by the Second and Third Authors. After removing, tissue specimens were immediately fixed in buffered formalin 10% at room temperature (RT) for 24 h. Then they were rinsed in phosphate buffered saline (PBS, pH 7.4), dehydrated in an ascending series of alcohol and embedded in paraffin via xylene. Subsequently, 3–5 μm serial sections were cut and processed for Haematoxylin/Eosin, Masson-Goldner Trichrome, Alcian Blu and immunohistochemical stainings.

Immunohistochemical experiments were performed using the indirect technique [35]. Sections were deparaffinised and endogenous peroxidase was blocked by incubation in 3% hydrogen peroxide for 5 min at RT. The following antibodies were used: mouse monoclonal anti-human ER-⍺ (1:100 titre, M7047; Dako - California, USA), rabbit polyclonal anti-human ER-β (1:100 titre, SC-8974; Santa Cruz Biotechnology - California, USA), mouse monoclonal anti-human PR (1:100 titre, M3568; Dako - California, USA); the vascularity parameter of the Bonar scale was investigated with a mouse monoclonal antibody anti-CD34 (1:100 titre, clone QBEnd/10 CM084B; Biocare - Italy). After washing with Tris-Buffered saline (TBS), sections were incubated with their primary antibody: ER-⍺, ER-β and PR for 2 h at 37 °C + o.n., CD34 for 2 h at RT. Preparations were then washed three times with TBS, and incubated with their secondary antibody following the kit protocol Dako EnVision Dual Link System-HRP: antimouse (EnVision FLEX LINKER mouse) for 15 min and anti-rabbit (EnVision FLEX/HRP) for 30 min. Sections were then washed three times with TBS and finally incubated in diaminobenzidine (DAB, Dako) for 5 min, followed by haematoxylin counterstaining.

Semiquantitative evaluation of immunoreactivity was performed at × 40 magnification in 10 microscopic fields randomly chosen by means of a whole slide scanner Hamamatsu Photonics Nanozoomer Digital Pathology Scanner 2.0RS. For each specimen, the expression of ER-⍺, ER-β, PR was quantified considering four grades of diffuse immunoreactivity: 0 = no expression; 1 = weak expression; 2 = high expression; 3 = very high expression. Immunoreactivity for sex steroid receptors was considered separately for tenocytes and vascular cells. Similarly, a semiquantitative analysis of tendinopathy was performed at × 4, × 10, × 20 magnification according to the Bonar histopathological scale [33, 34]. It considers four parameters: tenocytes, ground substance, collagen, vascularity. Each parameter is scored with a four-point scoring system, with 0 indicating healthy tissue and III highly degenerated tissue. The final score is comprised between 0 (normal tendon) and 12 (most severe abnormality detectable). Negative control slides processed without primary antibodies were included for each staining. All measurements were performed simultaneously by two observers without knowledge of the patient’s data, using a double-headed microscope. Intraobserver agreement was higher than 90%.

### Statistical analysis

Data are expressed as mean and standard deviation (±SD). Since the data showed an abnormal distribution, the statistics were performed using the Spearman correlation coefficient and the Mann-Whitney *U* test. Only *p* values < 0.05 were considered to be statistically significant. A priori power analysis was performed for sample size estimation, based on data from the study of Toesca et al. [21], comparing the ER-⍺ tissue expression between women and men. The effect size (ES) in this study was 2.5, considered to be extremely large according to Cohen’s (1988) criteria. With an alpha of 0.05 (two-tailed) and power of 0.80, the projected sample size needed with this effect size is approximately 8 for this between group comparison. Thus, our proposed sample size of 24 will be more than adequate for the main objective of this study.

## Results

**Fig. 2:**
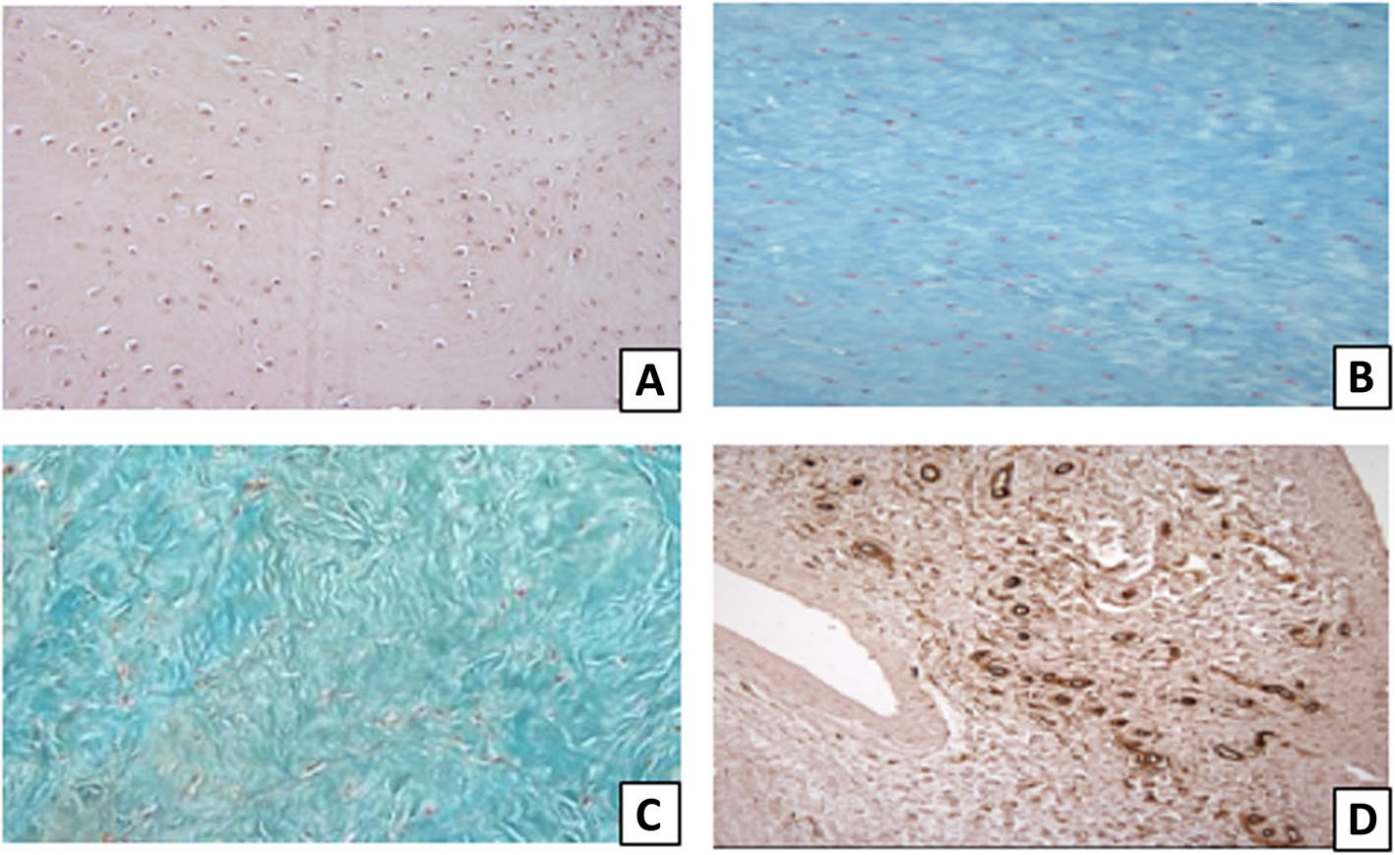
Histomorphological analysis of the supraspinatus tendon alterations following the Bonar Scale criteria. **A** Haematoxylin/Eosin showed pathological rounded nuclei and an increased cytoplasm in tendon cells. **B** Alcian Blu highlighted ground substance abnormalities with increased intrafascicular and interfascicular deposits of GAG. **C** Masson-Goldner Trichrome showed pathological collagen arrangement, with disorganization of fibre bundles. **D** CD34 immunohistochemical staining showed pathological neovascularization. Original magnification: × 200 (**A**), × 100 (**B**, **C**, **D**)

**Fig. 3:**
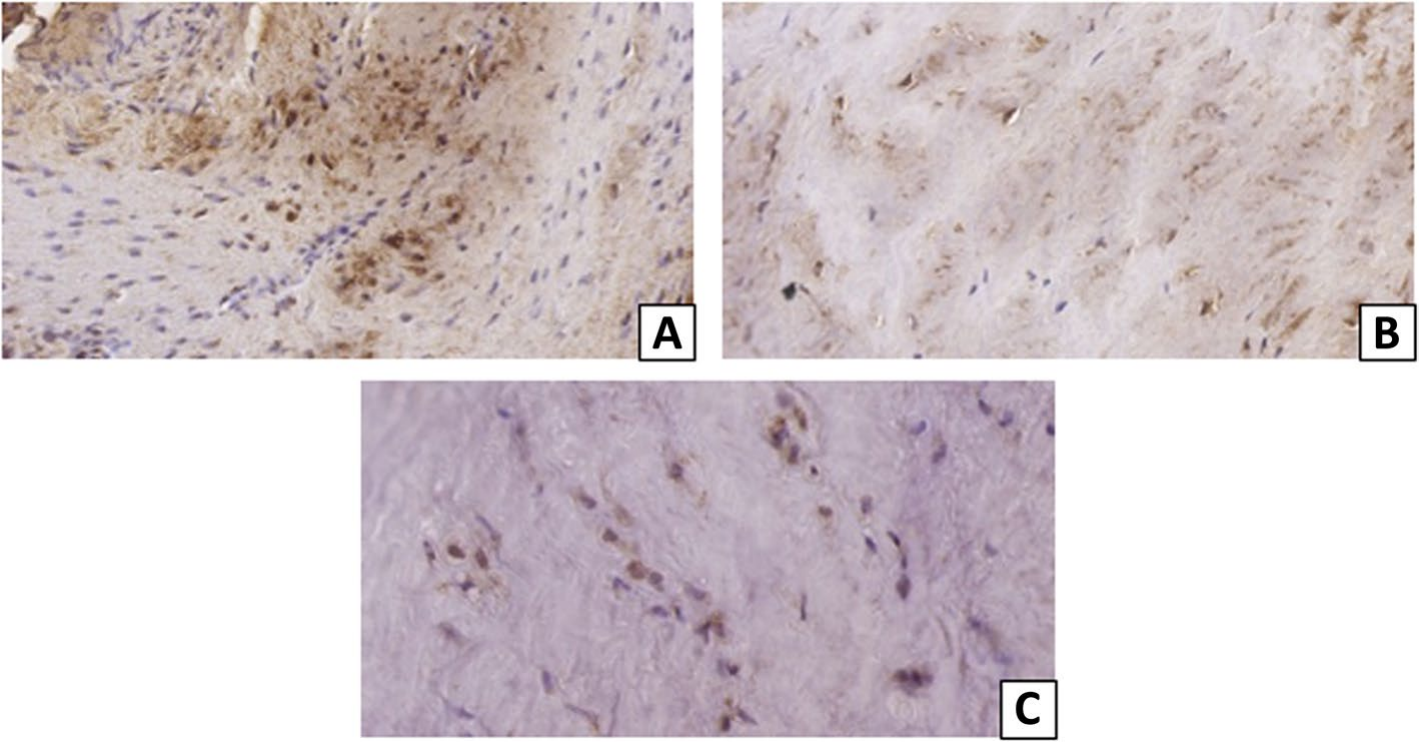
Immunohistochemical expression of ER-α, ER-β and PR in tenocytes of the supraspinatus tendon. **A** ER-α. **B** ER-β. **C** PR. Original magnification: × 40

**Fig. 4:**
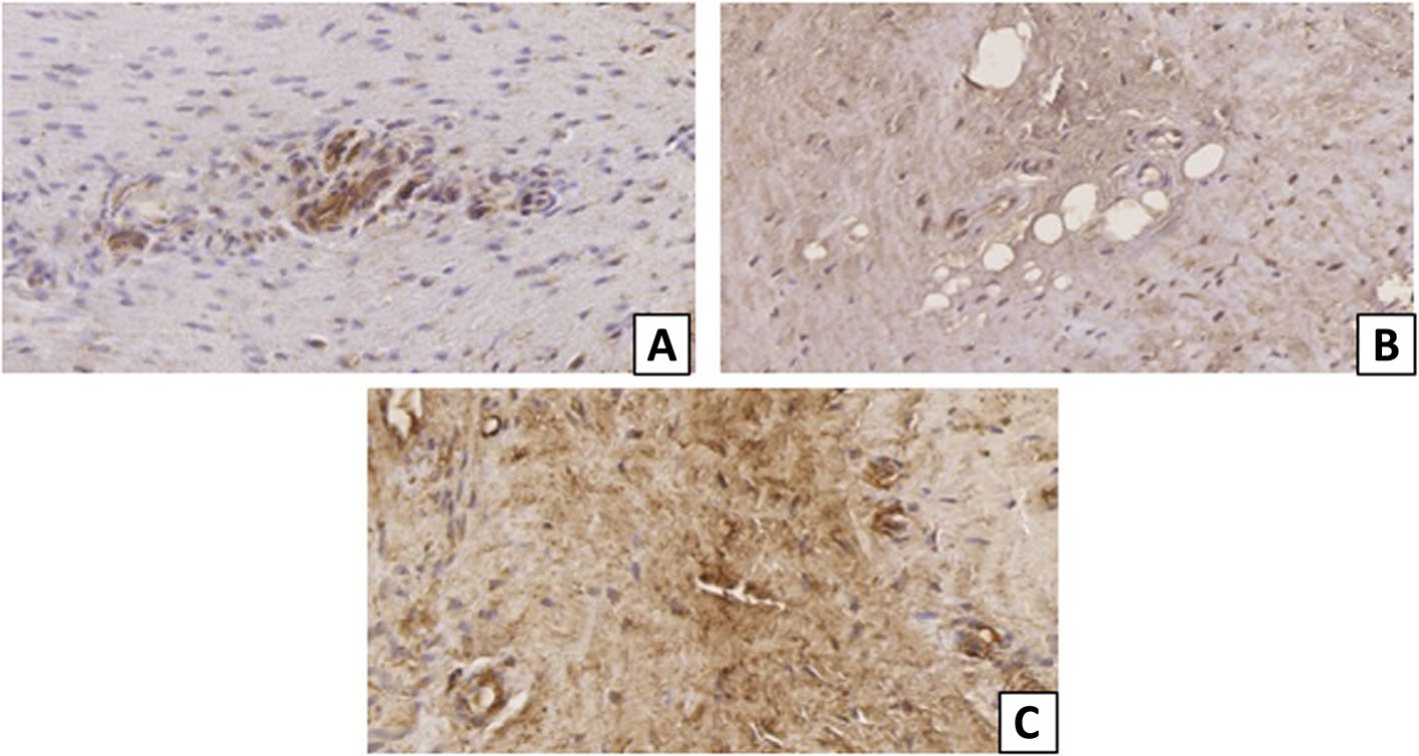
Immunohistochemical expression of ER-α, ER-β and PR in vascular cells of the supraspinatus tendon. **A** ER-α. **B** ER-β. **C** PR. Original magnification: × 40

Twenty-seven patients were initially included but 3 were excluded as they had previous surgery. The study enrolled 24 patients treated at our institution. The mean age of patients was 63.08 years (± 7.92, range 51–78). Bioptic samples of the supraspinatus tendon were collected from 15 women (mean age 63.93 ± 7.05) and 9 men (mean age 61.67 ± 9.02) undergoing surgery for RC tears. 27 patients were initially included but 3 were excluded as they had previous surgery. The total Bonar score of supraspinatus tendon specimens ranged from 2 to 10 (mean value 6.58 ± 1.96). In the female population, the mean value of the total Bonar score was 7.20 ± 1.51. In the male population, the mean value of the total Bonar score was 5.56 ± 2.17 (Fig. 2). Tissue specimens stained with Haematoxylin/Eosin showed pathological rounded nuclei and an increased cytoplasm in tendon cells, mainly presenting a grade II alteration of tenocyte morphology in men and women. The Alcian Blu staining highlighted ground substance abnormalities in tendon tissue, with increased intrafascicular and then interfascicular amount of glycosaminoglycans (GAG) disarraying collagen fibers. Grade II of ground substance alteration was mainly expressed in women, whereas men showed mainly a grade I. The Masson-Goldner Trichrome staining showed pathological collagen arrangement in tendinopathy, with loss of architecture and separation of fibre bundles. A grade II alteration of collagen arrangement was mainly observed in men and women. As regards vascularization, tendons showed occasional pathological clusters of capillaries. Grade I and grade III of alteration in vascularity was mainly observed in our female population. Men presented a higher expression of grade I (Tables 3 and 4). The expression of ER-⍺, ER-β and PR in the nuclei of tenocytes and vascular cells was evaluated separately for women and men. Both groups showed the expression of sex steroid receptors in the pathological supraspinatus tendon, with quantitative gender differences of immunoreactivity (Figs. 3 and 4). A four level (from 0 to 3) scoring system was adopted in order to quantify tissue immunoreactivity. In the female population, specimens showed high levels of ER-⍺ expression in the nuclei of tenocytes with a grade 3 mainly observed; men presented mainly a grade 1. The ER-⍺ positivity in the nuclei of vascular cells showed prevalently the expression of grade 0 for both men and women. Similarly, the nuclear expression of ER-β in tenocytes of the female group presented mainly a grade 3; men showed an equal expression of grades 1 and 2. The ER-β nuclear expression of vascular cells showed mainly a grade 0 for both men and women. The PR immunoreactivity in the nuclei of tenocytes showed mainly a grade 2 in the female group; men presented mainly a grade 1. The PR nuclear expression of vascular cells showed mainly a grade 0 for both men and women (Tables 5 and 6). Statistical analysis highlighted a significant gender difference of immunoreactivity for ER-β (*p* = 0.048) and PR (*p* = 0.004), with postmenopausal women showing markedly higher levels of expression than men. No statistically significant differences were found between males and females in ER-⍺ tendon expression (*p* = 0.055) (Table 7).

**Table 3:** Semiquantitative evaluation of tendon alterations in women according to the Bonar Scale

Variables	Grade 0	Grade 1	Grade 2	Grade 3
**Tenocytes**	0 (0%)	3 (20%)	8 (53.3%)	4 (26.7%)
**Ground substance**	2 (13.3%)	5 (33.3%)	7 (46.7%)	1 (6.7%)
**Collagen**	0 (0%)	0 (0%)	12 (80%)	3 (20%)
**Vascularity**	4 (26.7%)	5 (33.3%)	1 (6.7%)	5 (33.3%)

Data are absolute values and percentages of positively stained cells in women’s samples for the 4 parameters of the Bonar Scale: it allows a semiquantitative analysis of tendinopathy considering alterations of tenocytes, ground substance, collagen and vascularity. Each parameter is scored with a four-point scoring system, with 0 indicating healthy tissue and III highly degenerated tissue. The final score is comprised between 0 (normal tendon) and 12 (most severe abnormality detectable)

**Table 4:** Semiquantitative evaluation of tendon alterations in men according to the Bonar Scale

Variables	Grade 0	Grade 1	Grade 2	Grade 3
**Tenocytes**	1 (11.1%)	2 (22.2%)	5 (55.6%)	1 (11.1%)
**Ground substance**	3 (33.3%)	4 (44.4%)	2 (22.2%)	0 (0%)
**Collagen**	0 (0%)	3 (33.3%)	5 (55.6%)	1 (11.1%)
**Vascularity**	2 (22.2%)	5 (55.6%)	0 (0%)	2 (22.2%)

Data are absolute values and percentages of positively stained cells in men’s samples for the 4 parameters of the Bonar Scale

**Table 5:** Semiquantitative evaluation of ER-⍺, ER-β, PR expression in the supraspinatus tendon of women

Variables	Grade 0	Grade 1	Grade 2	Grade 3
**ER-⍺ tenocytes**	0 (0%)	3 (20%)	5 (33.3%)	7 (46.7%)
**ER-⍺ vessels**	9 (60%)	4 (26.7%)	1 (6.7%)	1 (6.7%)
**ER-β tenocytes**	0 (0%)	4 (26.7%)	5 (33.3%)	6 (40%)
**ER-β vessels**	8 (53.3%)	4 (26.7%)	3 (20%)	0 (0%)
**PR tenocytes**	0 (0%)	3 (20%)	8 (53.3%)	4 (26.7%)
**PR vessels**	8 (53.3%)	3 (20%)	3 (20%)	1 (6.7%)

Data are absolute values and percentages of positively stained cells in women’s samples for the expression of ER-⍺, ER-β, PR in the supraspinatus tendon: it was quantified considering four grades of diffuse immunoreactivity: 0 = no expression; 1 = weak expression; 2 = high expression; 3 = very high expression

**Table 6:** Semiquantitative evaluation of ER-⍺, ER-β, PR expression in the supraspinatus tendon of men

Variables	Grade 0	Grade 1	Grade 2	Grade 3
**ER-⍺ tenocytes**	0 (0%)	5 (55.6%)	3 (33.3%)	1 (11.1%)
**ER-⍺ vessels**	8 (88.9%)	1 (11.1%)	0 (0%)	0 (0%)
**ER-β tenocytes**	1 (11.1%)	4 (44.4%)	4 (44.4%)	0 (0%)
**ER-β vessels**	7 (77.8%)	2 (22.2%)	0 (0%)	0 (0%)
**PR tenocytes**	2 (22.2%)	5 (55.6%)	2 (22.2%)	0 (0%)
**PR vessels**	8 (88.9%)	1 (11.1%)	0 (0%)	0 (0%)

Data are absolute values and percentages of positively stained cells in men’s samples for the expression of ER-⍺, ER-β, PR in the supraspinatus tendon

**Table 7:** Gender difference of expression for ER-⍺, ER-β, PR in the supraspinatus tendon

	ER-⍺		ER-β		PR	
	Females	Males	Females	Males	Females	Males
Mean ± SD	2.3 ± 0.8	1.6 ± 0.7	2.1 ± 0.8	1.3 ± 0.7	2.1 ± 0.7	1 ± 0.7
N	15	9	15	9	15	9

Statistical analysis highlighted a significant gender difference of immunoreactivity for ER-β (*p* = 0.048) and PR (*p* = 0.004), with postmenopausal women showing markedly higher levels of expression than men. No statistically significant differences were found between males and females in ER-⍺ tendon expression (*p* = 0.055). Data are expressed as mean ± SD

There was a positive correlation between the expression of ER-⍺ and ER-β in women’s tendon (*r* = 0.59; *p* = 0.02). Similarly, there was a positive correlation between ER-β and PR in the same group (*r* = 0.72; *p* = 0.002). Moreover, a negative correlation in women between age and PR (*r* = − 0.56; *p* = 0.027) was found. Only in women, the ER-β expression positively correlated with the total Bonar histopathological score (*r* = 0.64, *p* = 0.01) and the ER-β vascular expression positively correlated with ground substance alterations (*r* = 0.62, *p* = 0.014). The same analysis performed in men did not show any statistically significant correlation.

## Discussion

Although the exact pathogenesis of RC tendinopathy is still under debate [5, 7], it is thought to be a multifactorial disease [3], with several cofactors that contribute to weaken tendons until their rupture. Previous studies have indicated that repetitive motion disorders occur mainly in women [14] and in advanced age [5, 6]. It may imply a potential role of postmenopausal hormonal modifications in influencing the natural history of these ailments.

This study showed that both men and women affected by RC tears express nuclear receptors for estrogens and progesterone in tendon cells. Immunoreactivity was found in tenocytes and in vascular cells of the same tissue. The presence of ER-⍺, ER-β and PR in RC tendons indicates that sex steroid hormones may influence the homeostasis of tendon tissue. Consistent with this role, several studies on rats have demonstrated a reduction in collagen content in joint capsules, skin, tendons and arteries after a long-term estrogenic treatment [36–38]. Moreover, in humans estrogens may influence ligament structure modulating the expression of collagen type I and III [23], fibroblast proliferation [25] and inflammatory pathways [39]. These and other hormonal effects may be responsible for structural modifications in tendon tissue, explaining the higher incidence of the disease in women.

It is known that ER-⍺ and ER-β, the main isoforms of estrogen receptor, can have mutual complex interactions leading to different effects on tissues. On the mammary gland, 17β-estradiol modulates different signaling pathways depending on the isoform of its receptor: the binding to ER-⍺ promotes cellular proliferation, whereas the binding to ER-β inhibits proliferation and favors differentiation [40, 41]. It means that different levels of receptor expression may lead to opposite biological effects on the same tissue. An ER-β overexpression has been found in tenocytes and synovial tissue of postmenopausal women with carpal tunnel syndrome, suggesting that ERs may play a role in its pathogenesis [42]. This study showed that ER-β and PR are markedly more expressed in postmenopausal women’s samples compared to their male counterpart. In contrast, ER-⍺ did not show a statistically significant difference of expression between males and females. Furthermore, only in women there was a positive correlation between the expression of ER-⍺ and ER-β and between ER-β and PR. These findings suggest that in torn supraspinatus tendons the ER-⍺/ER-β ratio is approximately 1, but undoubtedly further studies are required to clarify how ERs mutually influence their effects on this tissue.

In postmenopausal women, the ER-⍺ and ER-β expression in the supraspinatus tendon did not vary with age. In contrast, the PR expression decreased with age and it is consistent with data from other studies on the transverse carpal ligament [21]. The exact role of PR in the supraspinatus tendon remains unclear, however previous studies on rats have investigated how progesterone may influence the musculoskeletal system: it has been demonstrated that the collagen content of both hip and knee joint capsule is significantly decreased by estrogen or estrogen combined with progesterone [36]. The PR gene (PGR) is a typical ER target gene, therefore the PR expression has been historically used as a biomarker of the ER pathway on a tissue [43]. Many authors have studied how these mutual receptor interactions influence the natural history of breast cancer, wherein the PR expression is considered a positive prognostic factor [44, 45]. Indeed, in breast tissue progestogens (compounds that activate PR) exert an anti-proliferative effect [46] because PR sequesters ER away from its pro-proliferative gene targets, thereby inhibiting cancer growth [47]. Identification of progesterone effects on the RC tendons may be helpful to understand the interactions with estrogens on this tissue.

In order to investigate potential links between the expression of sex steroid receptor and tendinopathy, we performed a semiquantitative analysis of supraspinatus tendon specimens according to the criteria of the Bonar histopathological scale [33]. The Bonar scale allowed us to stratify patients for the severity of tendinopathy. Results showed a positive correlation in women between the ER-β expression and the total Bonar histopathological score. Furthermore, consistently with the rationale of the present research, it was evident only in the female population suggesting that gender differences may influence the pathogenesis of the disease.

Surprisingly, only ER-β showed correlations with the histopathological findings of RC tears. It implies that this receptor may play a major role in determining tendon alterations if compared to ER-⍺ and PR. Some authors have reported that the ER-β expression is associated with the severity of the De Quervain’s Syndrome, hypothesising that estrogens aggravate inflammation and angiogenesis through an increased ER-β expression [22]. Moreover, a study on rats has demonstrated that the coexistence of mechanical stress and estrogen deficiency exacerbates tendinopathy through up-regulating the ER-β-associated apoptosis in tenocytes [48]. In addition, several studies have reported that specific genetic variants of the Estrogen-Related Receptor Beta (ESRRB) gene convey a significantly increased risk of RC tearing and failure of RC healing compared to population controls [49–52]. Additional investigations are needed to clarify how changes in estrogens levels affect biological responses of the shoulder.

Specimens showed immunoreactivity for ER-⍺, ER-β and PR in vascular cells of the supraspinatus tendon, suggesting a potential role of sex hormones in angiogenesis. As a matter of fact, estrogens are known to promote bone angiogenesis [53] and to have vasodilator effects increasing microvascular permeability in the uterus [54–56]. It is possible that sex hormones fluctuations determine modification in blood supply to tendon tissue, altering its homeostasis and predisposing it to injures.

This study also reported a positive correlation between the ER-β vascular expression and ground substance abnormalities. The ER-β is believed to have roles in promoting cell survival in hypoxic environments [57, 58]: in fact it has been shown to interact with the Hypoxia-Inducible Factor (HIF), stimulating transcription of its target genes [57]. The insertional footprint of the RC is considered a relatively hypoxic microenvironment [59–61], justifying an elevated ER-β expression. Furthermore, studies performed on the RC and on the anterior tibialis tendon revealed that ground substance alterations (such as GAG overexpression and, consequently, fibrocartilagineous chondroid metaplasia) are a functional adaptation to persistent hypoxia in poorly vascularized areas of tendons [62, 63]. These findings may explain the relationship between ER-β, vascularization and ground substance alterations reported in the present study, however the role of this process in the pathogenesis of RC tears needs to be demonstrated.

This study has some limitations. Firstly, a relatively small sample size did not allow us to perform large-scale statistical analyses. Secondly, the sparseness of samples from patients less than 50 years old made it difficult to analyse potential modification of the sex steroid receptor expression before and after menopause in tendons. Thirdly, due to the lack of a control group we were unable to compare our findings with normal healthy tissue.

## Conclusions

This is the first study that reveals the expression of estrogen and progesterone receptors in the supraspinatus tendon of patients with RC tendinopathy. Results seem to suggest a potential link between this hormonal expression and the age, the gender and the severity of the typical histopathological alterations of the disease. As reported by several pieces of evidence from other works, the ER-β showed the most significant association with tendon abnormalities. It implies that this receptor may play a leading role in altering tendon homeostasis compared to ER-⍺ and PR. The present study tried to shed further light on the unclear pathogenesis of this ailment, suggesting a gender predisposition. However, considering the study design, the lack of a control group, and the lack of data before the tendon rupture, the hormone receptor expression cannot be directly linked to the pathogenesis of the rotator cuff rupture.

The localization of sex steroid hormone receptors in RC tendons is the first step to understand the potential role of estrogens and progesterone in predisposing shoulder injuries. Additional investigations should be conducted in order to evaluate how sex hormones and their receptors influence biological responses in the RC.

## Data Availability

The dataset supporting the conclusions of this article is included within the article.

## Abbreviations

RC: Rotator cuff
ER-⍺: Estrogen receptor-alpha
ER-β: Estrogen receptor-beta
PR: Progesterone receptor
MRI: Magnetic resonance imaging
RT: Room temperature
PBS: Phosphate buffered saline
TBS: Tris-buffered saline
DAB: Diaminobenzidine
SD: Standard deviation
GAG: Glycosaminoglycans
PGR: Progesterone receptor gene
ESRRB: Estrogen-Related Receptor Beta
HIF: Hypoxia-Inducible Factor
